# Sporting activity does not fully prevent bone demineralization at the impaired hip in athletes with amputation

**DOI:** 10.3389/fphys.2022.934622

**Published:** 2022-10-19

**Authors:** Valentina Cavedon, Marco Sandri, Ilaria Peluso, Carlo Zancanaro, Chiara Milanese

**Affiliations:** ^1^ Laboratory of Anthropometry and Body Composition, Department of Neurosciences, Biomedicine and Movement Sciences, University of Verona, Verona, Italy; ^2^ Council for Agricultural Research and Economics (CREA-AN), Research Centre for Food and Nutrition, Rome, Italy

**Keywords:** paralympic athletes, sitting volleyball, amputee soccer, DXA, bone health, osteoporosis, above-knee amputation, below-knee amputation

## Abstract

There is lack of information about bone mineralization at the lumbar spine and bilateral hips of athletes with unilateral lower limb amputation. The present study assessed for the first time the areal bone mineral density at the lumbar spine and at the hip of the able and impaired leg by means of Dual-Energy X-Ray Absorptiometry using a large sample (*N* = 40) of male athletes. Results showed that bone demineralization in athletes with unilateral lower limb amputation is found at the impaired hip but not at the lumbar spine and may therefore be site-specific. The extent of hip demineralization was influenced by the level of amputation, with about 80% of athletes with above knee amputation and 10% of athletes with below knee amputation showing areal bone mineral density below the expected range for age. Nevertheless, a reduced percentage of fat mass and a lower fat-to-lean mass ratio in the residual impaired leg as well as a greater amount of weekly training was positively associated with bone mineralization at the impaired hip (partial correlation coefficients = 0.377–0.525, *p* = 0.040–0.003). Results showed that participation in adapted sport has a positive effect on bone health in athletes with unilateral lower limb amputation but is not sufficient to maintain adequate levels of bone mineralization at the impaired hip in athletes with above-knee amputation. Accordingly, physical conditioners should consider implementing sporting programs, according to the severity of the impairment, aimed at improving bone mineralization at the impaired hip and improve body composition in the residual impaired leg.

## Introduction

In the general population, the risk factors for bone demineralization are advanced age, female sex, low body weight, history of parental hip fracture, ethnic background (white people are at higher risk than black people), previous fracture due to a minor trauma, rheumatoid arthritis, current smoking and poor diet (i.e. alcohol excess, vitamin D deficiency and low calcium intake) (Cosman et al., 2014).

Bone is a remarkable tissue that is responsive to external stimuli (Rosa et al., 2015). According to the mechanostat theory of bone proposed by Frost (Frost, 1987; Frost, 2003), bone mass is a direct result of the mechanical use of the skeleton and regions of bone experiencing low mechanical loads are removed, while regions of bone experiencing high mechanical loads become consolidated. Bone formation, regeneration and degradation processes are stimulated by mechanical strain as a result of applied mechanical stress in the form of muscular contraction, impact loading and gravitational forces (Kini and Nandeesh, 2012). Bone loss due to mechanical unload occurs for example in some types of physical impairment like spinal cord injury (SCI) and lower limb amputation (LLA) (Sherk et al., 2008; Dionyssiotis et al., 2021). In SCI the greatest amount of bone loss takes place in the first 2 years after injury, after that bone loss occurs gradually and bone formation is inhibited (Ragnarsson and Sell, 1981; Demirel et al., 1998; Dauty et al., 2000; Eser et al., 2005). In people with LLA, the bone turnover rate seems to be affected by prolonged bed rest, disuse atrophy leading to decreased muscle mass and lack of muscular contraction during activity, reduced ambulation, lack of exercise, decreased vertical loading and changes in load distribution on the impaired lower limb (Davies and Datta, 2003; Kontulainen et al., 2004; Nawijn et al., 2005; Bukowski, 2006). Moreover, scientific data showed that in people with LLA, bone demineralization mainly occurs in the hip of the impaired lower limb (Leclercq et al., 2003; Sherk et al., 2008; Smith et al., 2011, 2009; Bemben et al., 2017), especially in those with an above-knee amputation (AKA). For these reasons, regardless of age and other risk factors, people with lower limb amputation (LLA) suffer from bone demineralization and, consequently, they are at risk of osteoporosis and fragility fractures (Leclercq et al., 2003; Sherk et al., 2008; Cavedon et al., 2021; Haleem et al., 2021).

Today, the Dual-Energy X-ray Absorptiometry (DXA) is the most widely used technique to assess areal bone mineral density (aBMD) (Kanis et al., 2008). The DXA-measured aBMD value is the single best predictor of fracture at each specific site and provides the measures on which the World Health Organization diagnostic categories are based (Kanis and Glüer, 2000). The hip and lumbar spine skeletal sites are considered of biological relevance for the assessment of bone demineralization as these are the most common sites for fragility fractures in the general population (Hans et al., 2006; Anderson et al., 2021). Today there are no specific guidelines for bone mineral density testing in people with LLA. In scientific literature (Sherk et al., 2008; Smith et al., 2011; Bemben et al., 2017) and in clinical practice, the choice methods and sites of measurement used in this population to assess bone health are based on recommendations for the general population from the International Society of Clinical Densitometry (The International Society for Clinical Densitometry, 2019).

Today, a growing number of people with LLA are involved in adapted sports like amputee soccer, wheelchair basketball, wheelchair tennis, sitting volleyball, track and field, para swimming, handbike and so on (World Amputee Football Federation, 2005; International Paralympic Committee, 2020). Exercise is one the primary modifiable factors associated with improved bone health outcomes (Weaver et al., 2016). In fact, in literature the osteogenic effect of exercise and its role in preventing or reversing bone loss at each life stage (Wiswell et al., 2002; Todd and Robinson, 2003; Cavedon et al., 2020; Milanese et al., 2021) is well established, while the lack of exercise has been shown to be associated with increased bone absorption (Kontulainen et al., 2001; Tanaka et al., 2001; Smeltzer et al., 2005). Moreover, it has been shown (Creighton et al., 2001; Milanese et al., 2014), that exercises which exert high-impact and weight loading on the skeleton (e.g., jumping) induce bone formation and enhance osteogenesis at the weight-bearing skeletal sites. To the best of our knowledge, in adapted sports for people with LLA the effects of mechanical loading forces on bone density at specific skeletal sites has never yet been investigated. As reported in able-bodied literature (Creighton et al., 2001; Carter and Hinton, 2014), weight bearing and skeletal loading can vary greatly between sports: in high impact loading sports (i.e., volleyball, basketball and gymnastic) or moderate impact loading sports (i.e., middle-to short-distance track and soccer) the ground reaction forces are greater than body weight, while in non-impact loading sports (i.e., swimming and cycling) the ground reaction forces are approximately equivalent to body weight (Stewart and Hannan, 2000; Rector et al., 2008; Carter and Hinton, 2014). In analogy with able-bodied literature (Creighton et al., 2001; Carter and Hinton, 2014), it is reasonable to assume that in several adapted sports the lower limb weight bearing and skeletal loading may be reduced or even lacking. Specifically, adapted sports involve muscle contraction forces especially on the upper body skeleton, but because body weight is supported either by a wheelchair (as with wheelchair basketball), a handbike or the court floor (as in the case of sitting volleyball), in these sports the gravitational loading components are considerably reduced or even lacking. Moreover, in other adapted sports (like amputee soccer), athletes do not wear a prosthesis during the game and, consequently, the muscle contraction in the impaired lower limb is reduced to a minimum. According to the above, it is reasonable to hypothesize that athletes with LLA are at risk of bone demineralization despite the practice of an adapted sport.

To date in the literature little is known about the bone health of athletes with LLA. In fact, only one study (Cavedon et al., 2021) assessed areal bone mineral density (aBMD) by-means of DXA in athletes with LLA showing that this athletic population have reduced aBMD in the impaired lower limb in comparison with age-matched able-bodied athletes. In this recent study, Cavedon and colleagues (Cavedon et al., 2021) measured aBMD at the whole-body and regional levels only (arms, trunk, and both limbs). Accordingly, data about aBMD assessed in specific skeletal sites (e.g., lumbar spine and hip) in athletes with LLA are still lacking. Consequently, research is needed to estimate aBMD at specific lower limb skeletal sites to understand whether this athletic population is at risk of bone demineralization. In addition, identifying any risk of bone demineralization and protective factors in athletes with LLA would be beneficial for designing programs aimed at improving bone health in this athletic population.

In order to fill these gaps in the scientific literature, this is the first study assessing aBMD by-means of DXA at specific skeletal sites (i.e., the lumbar spine and hips of both the non-impaired and impaired leg) in a large sample of male athletes with LLA. This study has a twofold aim. The first aim was to assess the possible extent of bone demineralization at specific skeletal sites according to the level of amputation (i.e., AKA and amputation below the knee [BKA]). As a second aim of this study, we explored the association between some unmodifiable (e.g., age, duration of amputation and age at amputation) and some modifiable factors (e.g., the amount of training and some DXA-measured body composition variables) with aBMD measured in the hip of the impaired leg. Finally, in noticing that athletes with LLA frequently have metal implants in the hip of the impaired leg, thereby preventing the measurement of bone parameters at that skeletal site, we tried to identify some predictors for aBMD at the hip of the impaired leg. We hypothesized that athletes with AKA only are at risk for bone demineralization at the hip of the impaired leg despite the practice of an adapted sport. However, we also expect that a greater amount of training and an improved body composition may mitigate bone demineralization at the hip of the impaired leg in athletes with LLA.

## Materials and methods

### Participants

To avoid the confounding effect of sex, only male athletes were included. Inclusion criteria to participate in this study were: white race, Caucasian ethnicity, age 18 or over and practicing an adapted sport at a competitive level for at least 1 year prior to testing. Exclusion criteria were: hemipelvectomy or hip disarticulation, ankle disarticulation or partial foot amputation, underweight status, previous fragility fractures and family history of fragility fractures, suffering from any chronic or systemic disease or other physical impairments, apart from the amputation, that might affect body composition or bone metabolism [i.e., tumors, rheumatoid arthritis, diabetes mellitus, osteogenesis imperfecta, untreated long-standing hyperthyroidism, chronic renal disease (grade 3 or higher) or chronic liver disease] and use of medications that might affect bone metabolism (i.e., steroid medication of ≥ 5 mg/day for ≥ 3 months).

Forty male athletes with LLA aged 36.9 ± 9.3 years were enrolled in this study. Athletes were split into two groups according to the level of amputation, i.e., athletes with AKA (*n* = 15) and athletes with BKA (*n* = 25). The origin of amputation was acquired in 38 athletes and congenital in 2 cases. The average duration of amputation was 12.9 ± 8.7 years and the average age at which amputation occurred was 12.8 ± 8.7 years. All athletes wore their prostheses more than 8 h a day and none used any other ambulatory aids. Five athletes were smokers and the average daily cigarette smoking was 8.6 ± 4.8. For all athletes the alcohol consumption was less than 3 units per day (equivalent to three or less standard drinks). Athletes had been practicing their particular sport for 6.48 ± 5.18 years and they were regularly training for 4.6 ± 1.9 h per week. The sports of the athletes enrolled in the study were para triathlon (*n* = 1), amputee soccer (*n* = 10), wheelchair basketball (*n* = 4), para cycling (*n* = 4), para shooting (*n* = 1), sitting volleyball (*n* = 16), para climbing (*n* = 3), para ice hockey (*n* = 1).

The study was conducted in accordance with the Declaration of Helsinki, and the protocol was approved by the Institutional Review Board of the local University. All the participants were informed about the aims of the study and the experimental procedures, and they knew that they could withdraw at any time. All participants read and signed the informed consent form.

### Testing procedures

Testing took place on the same day, in the late morning/early afternoon, after a 3 h–4 h fast. All participants were asked not to undertake any strenuous physical activity the day before each measurement session, and they were also required not to undertake any exercising on the day of the measurements.

#### Face-to-face questionnaire

All athletes completed a face-to-face questionnaire to confirm the participants’ eligibility criteria and to collect information about demographics, level of amputation, duration of amputation, the age at which amputation occurred, cigarette smoking, units per day of alcohol consumption, adapted sport practiced, years of sport experience at a competitive level and the amount of training expressed in hours per week. Athletes were also asked to report if their amputation was congenital or acquired and if they use ambulatory aids other than the prosthesis (e.g., crutches, walker, wheelchair).

#### DXA measurements

Body composition (fat mass and lean soft tissue mass) and aBMD were measured using DXA with the QDR Horizon (Hologic MA, United States; fan-beam technology, software for Windows 7 Professional). In our laboratory quality control of the DXA scanner is performed at least once weekly and before actual use by means of an encapsulated spine phantom (Hologic Inc., Bedford, MA, United States) to document the stability of DXA performance (Lewiecki et al., 2004).

Densitometry measurements were performed by the same trained operator according to the manufacturer’s procedures and consisted in one whole-body scan followed by one lumbar spine scan in a posteroanterior projection and two hip scans (one per leg). Prior to scanning, participants were asked to void their bladder and to remove all metal, jewellery or reflective material, including prostheses. During all scanning participants were clothed with a light covering (i.e. underwear). Body mass and stature, which are required by the DXA software to enable scanning, were assessed as follows. Body mass was assessed with prosthesis to the nearest 0.1 kg using certified electronic scale (Tanita electronic scale BWB-800 MA, Wunder SA.BI. Srl, Milan, Italy). The weight of the prosthesis was then measured and subtracted from the previous body mass measurement to get the actual body mass. Standing height was measured with prosthesis to the nearest 0.1 cm using a Harpenden portable stadiometer (Holtain Ltd., Crymych, Pembs. United Kingdom) according to conventional criteria and measuring procedures (Lohman and Martorell, 1988).

During the whole-body scan, positioning aids to support the impaired lower limb were employed to ensure there was no movement during the scans. During scanning, the impaired leg was rotated internally and abducted slightly to bring the femoral neck parallel to the scan table and to prevent foreshortening of the femoral neck.

**FIGURE 1:**
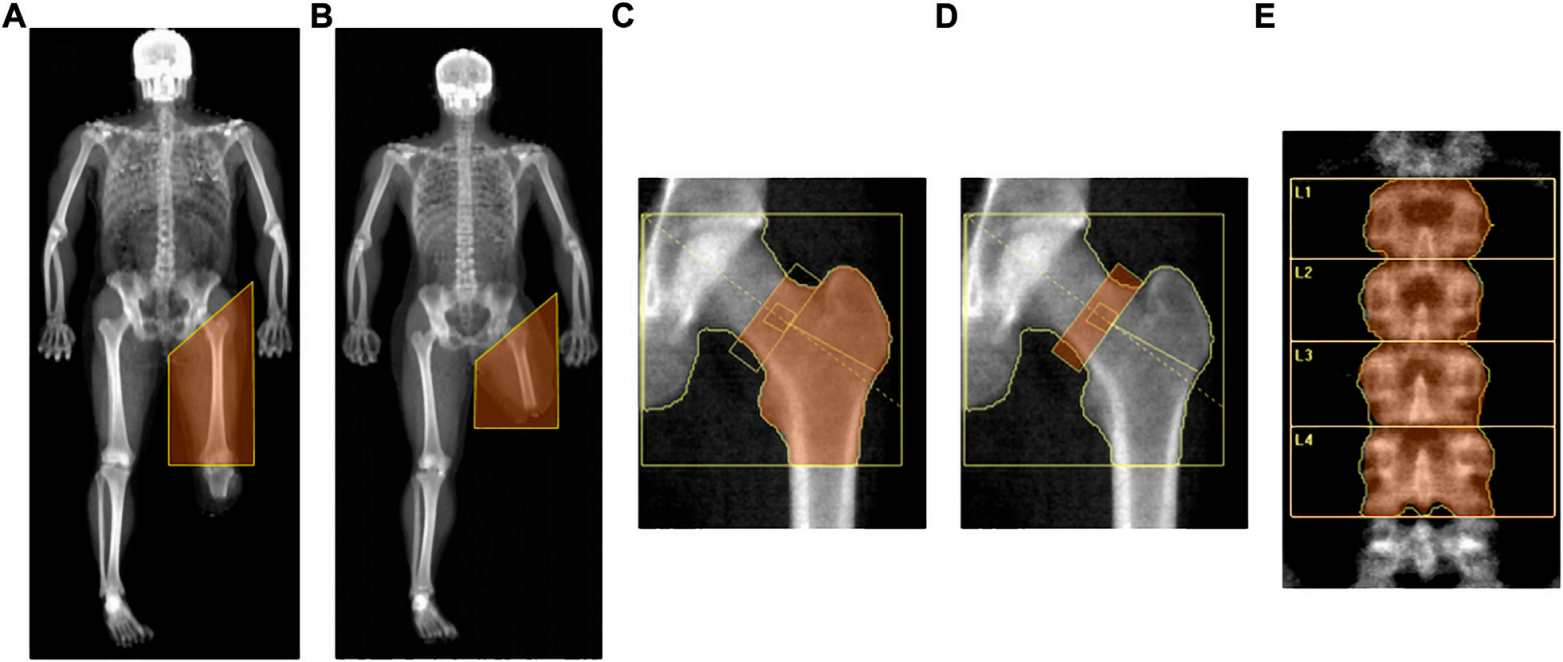
DXA-measured regions of interest., Legend. **(A)** Thigh region of the impaired leg assessed in athletes with AKA (whole-body scan); **(B)** Thigh region of the impaired leg assessed in athletes with BKA (whole-body scan); **(C)** Proximal femur region (hip scan); **(D)** Neck of the femur (hip scan); **(E) **Lumbar spine.

Analysis of DXA scans was performed by the same trained investigator to ensure consistency. From the whole-body scan, the thigh region of the impaired leg was delineated according to Hart and colleagues (Hart et al., 2015) by a proximal boundary formed by an oblique line passing through the femoral neck to a distal boundary formed by the horizontal line passing through the “tibial plateau” (i.e., the space between the femoral and tibial condyles) in athletes with BKA (Figure 1A), and by a horizontal line passing beyond the distal margin of the stump in the case of AKA (Figure 1B), which were used to assess fat and fat free mass. In line with previous literature (Cavedon et al., 2021), due to the differences in body mass among subjects with different levels of amputation, as a consequence to one segment being longer or shorter, only variables expressed in relative terms (i.e., variables expressed as a proportion of the whole like percentages or indexes) were considered for analysis. According to Cavedon and colleagues (Cavedon et al., 2021), at both the whole-body level as well as in the thigh region of the impaired leg, we included in the analysis the percentage of fat mass (% FM) as an index of adiposity and the fat-to-lean mass ratio (FM/LM) as an index of muscularity regardless of bone mineralization.

Today there are no specific guidelines for testing bone mineral density in people with LLA. In scientific literature (Sherk et al., 2008; Smith et al., 2011; Bemben et al., 2017) as well as in clinical practice, the choice of methods and sites of measurement used in this population to assess bone health are mainly based on recommendations for the general population from the International Society of Clinical Densitometry (The International Society for Clinical Densitometry, 2019). Accordingly, for men aged > 50 years with a disease or condition associated with bone loss (The International Society for Clinical Densitometry, 2019) and in line with previous literature on people with LLA (Sherk et al., 2008; Smith et al., 2011; Bemben et al., 2017), the aBMD values were expressed as grams of mineral per square centimetre (g/cm^2^) and were measured at both the hip skeletal site (regions of interest: total proximal femur and femoral neck) and the posteroanterior lumbar spine skeletal site (region of interest: lumbar vertebrae numbers 1 to 4 inclusive). Due to the purposes of this study, aBMD was evaluated at both hip skeletal sites and the non-impaired hip was used as the control.

For the hip scan, the Hologic’s standard global region of interest box was defined according to Feit and colleagues (Feit et al., 2020) by four lines placed 5 cm superior to the head of the femur, 5 cm medial to the head of the femur, lateral to the greater trochanter and 1 cm below the base of the lesser trochanter (Figures 1C,D). The total proximal femur region included the femoral neck, greater trochanter, intertrochanteric region and part of the femoral shaft (Figure 1C). The standard femoral neck region was a rectangular box of 1.5 cm × 4.9 cm anchored to the great trochanter (Feit et al., 2020) (Figure 1D). The operator checked that the femoral neck box did not contain any portion of the greater trochanter or the ischium (Figure 1D).

For the standard lumbar spine scan, the spine was centred in the densitometric image with roughly equal amounts of soft tissue on both sides, parallel to the sides of the image and included a part of both the thoracic spine with a vertebra with ribs and the upper part of the pelvis thereby making the iliac crest visible (Figure 1E). The standard global region of interest box with a width of 11.6 cm was placed with the top edge in correspondence to the upper margin of the first lumbar vertebra and the bottom edge in correspondence to the lower margin of the fourth lumbar vertebra (Figure 1E). The aBMD values were evaluated for lumbar vertebrae numbers 1 to 4 inclusive (Figure 1E).

The precision error (i.e., the percent coefficient of variation with repositioning) and the least significant change at 95% confidence interval (i.e., the least amount of BMD change that can be considered statistically significant) were calculated measuring 30 patients consecutively twice, repositioning the patient after each scan (The International Society for Clinical Densitometry, 2019). In our lab, the root mean square standard deviation and the least significant change were respectively 0.01 g/cm^2^ and 0.02 g/cm^2^ for the total hip region, 0.03 g/cm^2^ and 0.04 g/cm^2^ for the total spine region and 0.46 g/cm^2^ and 1.272 for the % FM at the whole-body level.

The recent Recommendations and Positions of the ISCD (The International Society for Clinical Densitometry, 2019; Anderson et al., 2021), suggest that for men younger than 50 years the Z-score should be used to interpret the DXA-measured aBMD results. Accordingly, a Z-score of −2.0 or lower was defined as below the expected range for age, and a Z-score above −2.0 was considered within the expected range for age (The International Society for Clinical Densitometry, 2019).

### Statistical analysis

Normality of data was assessed using the Kolmogorov-Smirnov test and descriptive statistics (mean ± standard deviation) were computed for all variables using standard procedures.

The two-tailed Student *t*-test for independent samples was carried out to compare means between the AKA e the BKA groups. Cohen’s d (d) was used to calculate the effect size and was interpreted according to Cohen (Cohen, 1988) as small (d = 0.2), medium (d = 0.5), and large (d = 0.8).

The Chi Square Test (*X* ^2^) was used to determine the association between the prevalence of aBMD values below the expected range for age and the level of amputation (AKA versus BKA).

A mixed-design ANOVA comparing side (Impaired leg versus Non-impaired leg) by level of amputation (AKA versus BKA) was conducted for the aBMD values measured at the total proximal femur and at the neck of the femur. For each ANOVA model, if significant interactions were detected (side by level of amputation) post hoc analysis with Bonferroni’s correction was carried out. Cohen’s partial eta squared (ƞ_p_^2^) was used to calculate the effect size in the mixed-design ANOVA. According to Cohen’s guidelines (Cohen, 1988), the effect size values were interpreted as small (ƞ_p_^2^ = 0.01), medium (ƞ_p_^2^ = 0.06), and large (ƞ_p_^2^ = 0.14).

The degree of association between two continuous variables, accounting for the level of injury (AKA and BKA), was measured by partial correlation (r_PC_). The partial correlation coefficient was considered small (r_PC_ = 0.00–0.30), moderate (r_PC_ = 0.31–0.49), large (r_PC_ = 0.50–0.69), very large (r_PC_ = 0.70–0.89), and almost perfect (r_PC_ = 0.90–1.00) as suggested by Hopkins (Hopkins, 2016).

Separate stepwise multiple regression analyses (enter, F < 0.05; remove, F > 0.1) were run using the aBMD values, measured at the hip scan of the impaired leg, as the dependent variable. The independent variables included in the model as potential predictor/s were the general characteristics of athletes (i.e., age, level of amputation [AKA and BKA], duration of amputation, age at amputation, sport experience and amount of training) and the aBMD values assessed at the hip scan of the non-impaired leg. Adjusted coefficients of determination (*R* ^2^) and standard error of the estimate (SEE) were used to represent the goodness-of-fit of the predictor model. The Durbin-Watson test was used to test for autocorrelation in the residuals, while the variance inflation factor was calculated to test collinearity. For both the regression models, Cohen’s f squared (f^2^) was used to calculate the effect size of the regression model and interpreted as small (f^2^ ≥ 0.02), medium (f^2^ ≥ 0.15) and large (f^2^ ≥ 0.35) according to Cohen’s guidelines (Cohen, 1988).

All analysis was performed with SPSS v. 26.0 (IBM Corp., Armonk, NY, United States) and the statistical significance was set at *p* ≤ 0.05.

## Results

The general characteristics of the AKA and BKA groups are reported in Table 1. No statistically significant differences were found between the two groups in age, duration of amputation, age at amputation, sport experience, amount of training, and the %FM and FM/LM at the whole-body level (*p* > 0.05 for all; Table 1). The two-tailed Student t-test for independent samples showed a statistically significant difference between the AKA and BKA groups in the %FM and FM/LM measured in the impaired thigh region (Table 1).

**TABLE 1:** General characteristics of the AKA and BKA groups. Data are mean ± standard deviation.

	AKA (*N* = 15)		BKA (*N* = 25)		*T*-test for independent samples		
Variables	Mean	SD	Mean	SD	t value	*p* value	d
Age (years)	36.8	11.0	36.9	8.4	−0.039	0.969	0.012
Duration of amputation (years)	12.9	6.7	12.9	9.9	−0.005	0.996	0.002
Age at amputation (years)	23.9	11.4	24.0	10.7	−0.030	0.976	0.010
Sport experience (years)	6.2	3.7	6.6	6.0	−0.257	0.799	0.089
Amount of training (hours per week)	4.1	1.6	4.9	2.1	−1.283	0.207	0.433
%FM at the whole-body level	24.5	6.5	23.5	5.4	0.554	0.583	0.177
FM/LM at the whole-body level	0.35	0.12	0.33	0.10	0.608	0.547	0.195
%FM in the impaired thigh region	35.4	8.7	27.5	5.6	3.350	0.002	1.073
FM/LM in the impaired thigh region	0.58	0.18	0.40	0.11	3.752	0.001	1.203

Legend. AKA, athletes with above-knee amputation; BKA, athletes with below-knee amputation; SD, standard deviation; d, Cohen’s d; %FM, percentage of fat mass; FM/LM, fat-to-lean mass ratio.

The DXA-measured variables of interest at the lumbar spine scan and at the hip scan performed in the non-impaired leg were obtained for all the participants (*N* = 40). Nine athletes had metal implants in the hip of the impaired leg and, accordingly, the variables of interest at this skeletal site were available for 10 athletes in the AKA group and for 21 athletes in the BKA groups (age: 35.0 ± 12.1 and 37.0 ± 8.8; duration of amputation: 14.5 ± 7.1 and 12.6 ± 9.5; age at amputation: 20.5 ± 12.0 and 24.4 ± 11.2; sport experience: 5.8 ± 3.9 and 6.1 ±; 5.5; volume of training: 4.2 ± 1.9 and 5.1 ± 2.2; respectively). No statistically significant differences were found between the two groups in the above-mentioned variables (*p* > 0.05 for all).

### Prevalence of skeletal aBMD values below the expected range for age according to the level of amputation

As reported in Table 2, all athletes in the BKA (*N* = 25) group had aBMD values above the expected range for age according to the ISCD criteria in any of the considered skeletal sites (Table 2). Similarly, all athletes in the AKA group (*N* = 15) had aBMD values above the expected range for age according to the ISCD criteria at the lumbar spine and at the non-impaired leg. Based on the ISCD criteria, 8 athletes out of 10 in the AKA group and 2 athletes out of 21 in the BKA group had a value of aBMD at the total proximal femur site of the impaired leg below the expected range for age (Table 2). The chi-square test of independence showed that such proportion is statistically significant, *X* ^*2*^ (1, *N* = 31) = 15.397, *p* < 0.001. Similarly, 6 athletes out of 10 in the AKA group had a value of aBMD at the total proximal femur site of the impaired leg below the expected range for age, while all athletes in the BKA group had an aBMD value above the expected range for age (Table 2). The chi-square test of independence showed that there was a statistically significant association between the level of amputation and the prevalence of aBMD values below the expected range for age, *X* ^*2*^ (1, *N* = 31) = 15.624, *p* < 0.001.

**TABLE 2:** Skeletal aBMD variables and prevalence of athletes with a Z-score’s value below the expected range for age according to the level of amputation.

	AKA						BKA					
	N	aBMD		Z-score			N	aBMD		Z-score		
Skeletal site		Mean	SD	Media	SD	Prevalence		Mean	SD	Media	SD	Prevalence
Lumbar spine	15	1.06	0.09	0.05	0.86	0%	25	1.07	1.00	−0.08	0.92	0%
Non-impiared leg												
Total proximal femur	15	0.99	0.11	−0.02	0.87	0%	25	1.04	0.14	0.22	0.78	0%
Neck of the femur	15	0.88	0.13	0.01	1.03	0%	25	0.92	0.15	0.27	0.90	0%
Impaired leg												
Total proximal femur	10	0.62	0.13	−2.58	0.97	80%	21	0.86	0.13	−1.00	0.81	10%
Neck of the femur	10	0.64	0.16	−1.73	1.31	60%	21	0.84	1.00	−0.23	0.83	0%

Legend. AKA, athletes with above-knee amputation; BKA, athletes with below-knee amputation; aBMD, areal bone mineral density; N, number of subjects with a valid DXA, scan; SD, standard deviation; Prevalence, prevalence of athletes with a value of a Z-score’s value below the expected range for age according to the Recommendations and Positions of the International Society of Clinical Densitometry.

### Difference in the aBMD and Z-score values at the hip of the impaired leg versus the non-impaired leg according to the level of amputation

**FIGURE 2:**
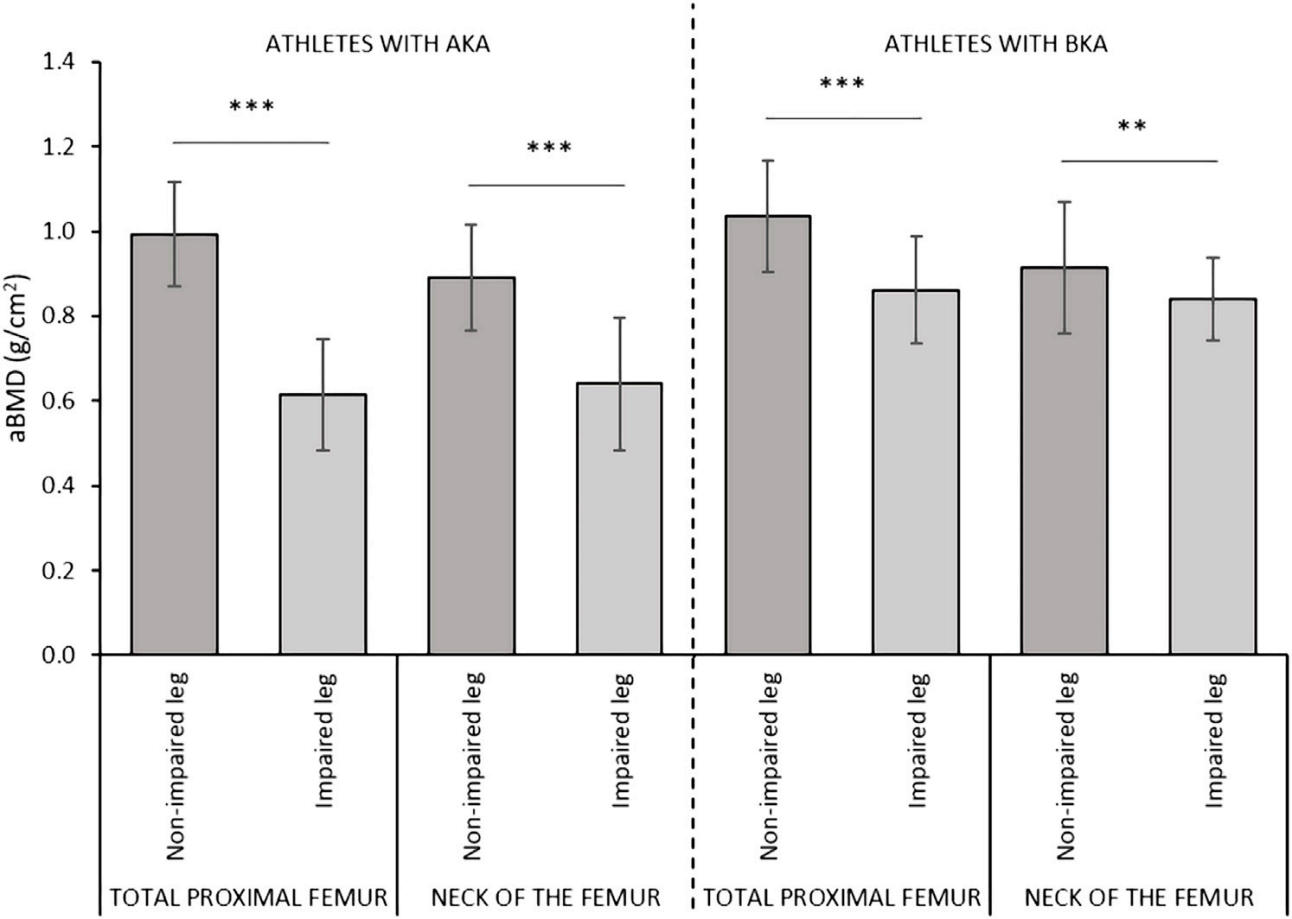
Comparisons of the aBMD values between the impaired and non-impaired leg according to the level of amputation. Data are presented as mean ± standard deviation. Legend. aBMD, areal bone mineral density; AKA, above-knee amputation; BKA, below-knee amputation. *p ≤ 0.05; **p ≤ 0.01; ***p ≤ 0.001.

**FIGURE 3:**
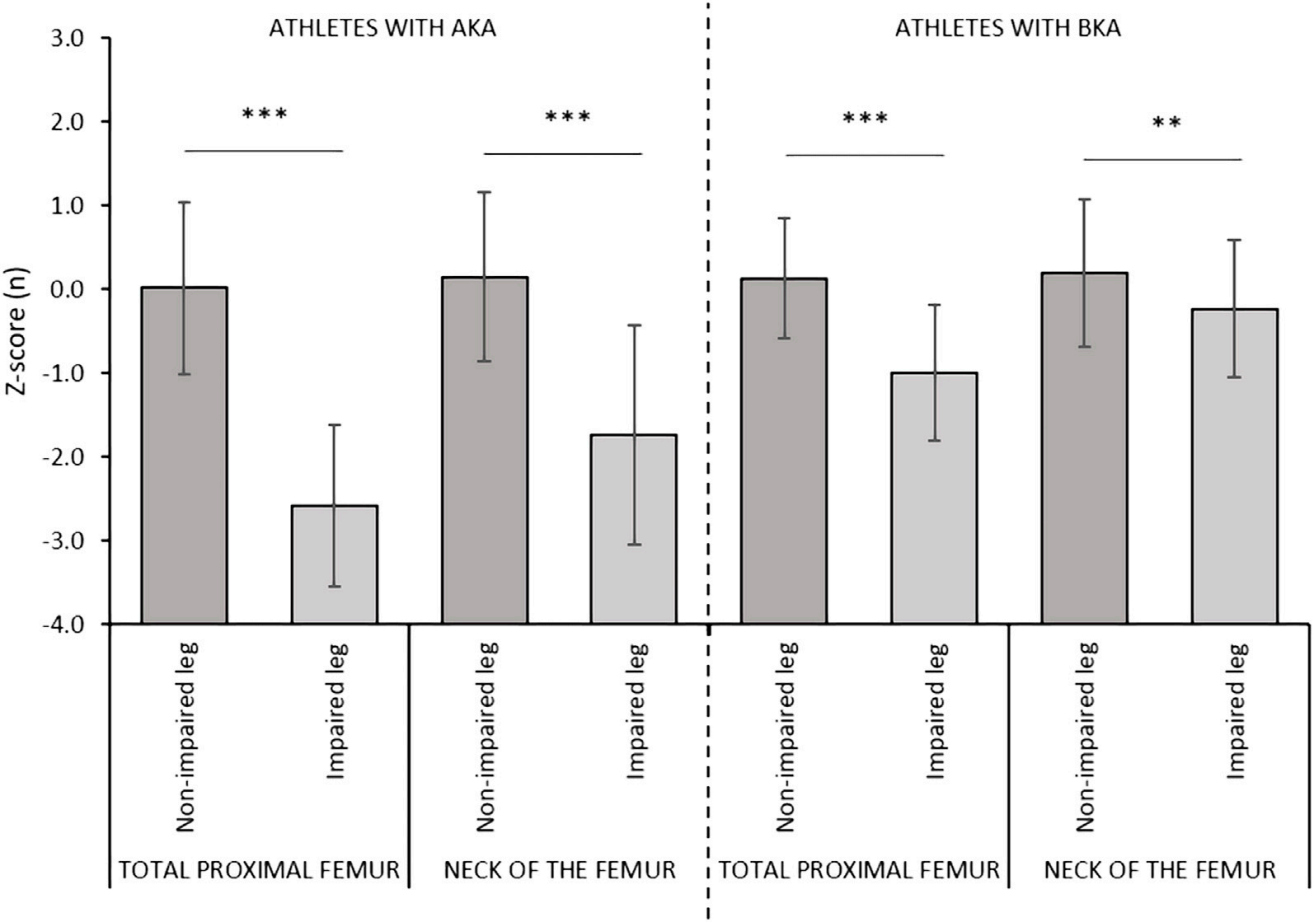
Comparisons of the Z-scores values between the impaired and non-impaired leg according to the level of amputation. Data are presented as mean ± standard deviation. Legend. AKA, above-knee amputation; BKA, below-knee amputation. *p ≤ 0.05; **p ≤ 0.01; ***p ≤ 0.001.

A mixed-design ANOVA comparing side (impaired leg versus non-impaired leg) by level of amputation (AKA versus BKA), showed a statistically significant effect of side by level of amputation for the aBMD and the Z-scores values measured at the total proximal femur (F = 42.747, *p* < 0.001, ƞ_p_^2^ = 0.596 and F = 46.726, *p* < 0.001, ƞ_p_^2^ = 0.617, respectively) and at the neck of the femur (F = 19.670, *p* < 0.001, ƞ_p_^2^ = 0.404 and F = 30.167, *p* < 0.001, ƞ_p_^2^ = 0.510, respectively). In the AKA group, the aBMD and the Z-score values were significantly lower at both the total proximal femur (−0.378 g/cm^2^ and −2.6, respectively) and at the neck of the femur of the impaired leg (−0.251 g/cm^2^ and −1.8, respectively) versus the non-impaired leg (Figures 2, 3). Also, in the BKA group, the aBMD and the Z-score values were significantly lower at both the total proximal femur (−0.173 g/cm^2^ and −1.1, respectively) as well as at the neck of the femur of the impaired leg (−0.074 g/cm^2^ and −0.4, respectively) versus the non-impaired leg (Figures 2, 3).

### Association between general characteristics and body composition, and the DXA-measured variables at the hip scan of the impaired leg accounting for the level of amputation

Aftercontrolling for the effect of the level of amputation, the duration of amputation, as well as the % FM and FM/LM measured at the whole-body level and in the thigh region of the impaired leg were moderately and negatively associated with the aBMD values assessed at the neck of the femur of the impaired leg (Table 3). A negative and statistically significant association was also found between the %FM measured at the thigh of the impaired leg and the Z-score assessed at both the total proximal femur and the neck of the femur regions of the impaired leg, as well as between the FM/LM measured at the thigh region of the impaired leg and the Z-score assessed at the neck of the femur of the impaired leg. Partial correlation analysis also showed a positive and statistically significant association between the amount of training and the aBMD measured both at the total proximal femur and the neck of the femur regions and with the Z-score assessed at the proximal femur only.

**TABLE 3:** Partial correlation coefficients (r_PC_) between general characteristics and body composition, and the DXA-measured variables at the hip scan of the impaired leg, accounting for the level of amputation (above-knee or below-knee amputation).

	Proximal femur		Neck of the femur	
Variables	aBMD	Z-score	aBMD	Z-score
Age	−0.187	0.016	−0.329	0.053
Duration of amputation	−0.229	−0.245	−0.359	−0.315
Age at amputation	0.013	0.202	-0.010	0.288
Sport experience	0.010	0.034	−0.126	−0.082
Amount of training	0.485**	0.399*	0.525**	0.354
%FM at the whole-body level	−0.325	−0.244	−0.486**	−0.261
FM/LM at the whole-body level	−0.307	−0.225	−0.464**	−0.232
%FM in the impaired thigh region	−0.338	−0.377*	−0.462**	−0.438*
FM/LM in the impaired thigh region	−0.321	−0.353	−0.447*	−0.416*

Legend. r_PC_, partial correlation coefficient; aBMD, areal bone mineral density; % FM, percentage of fat mass; FM/LM, fat-to-lean mass ratio; *, *p* ≤ 0.05; **, *p* ≤ 0.01; *p* ≤ 0.001.

Statistically significant correlations are in bold.

### Predictability of the aBMD values measured at the hip of the impaired leg

In the whole sample (*N* = 31), by entering age, level of amputation (AKA and BKA), duration of amputation, age at amputation, sport experience, amount of training and the aBMD values measured at the hip of the non-impaired leg as potential predictors in stepwise multiple linear regression analysis, a statistically significant model (F = 45.440 and *p* < 0.001) was estimated for aBMD at the total proximal femur of the impaired leg. The final model equation was:

aBMD at the total proximal femur=0.867(aBMD assessed at the total proximal femur of the non−impaired leg)+0.2(level of amputation, AKA=0 and BKA=1)+0.003 (age at amputation)−0.303. (1)

Adjusted *R* ^2^, SEE and f^2^ were 0.82, 0.07 and 4.43, respectively. The Durbin-Watson was 2.07, indicating that there was no autocorrelation between the residuals. The variance inflation factor was < 1.1 for all the predicting variables showing that multicollinearity between the variables in the model was weak.

In the whole sample (*N* = 31), by entering age, level of amputation (AKA and BKA), duration of amputation, age at amputation, sport experience, amount of training and the aBMD values measured at the non-impaired leg as potential predictors in stepwise multiple regression analysis, a statistically significant model (F = 27.403 and *p* < 0.001) was estimated for aBMD at the neck of the femur of the impaired leg. The model was:

aBMD at the neck of the femur=0.18(level of amputation, AKA=0 and BKA=1)+0.556(aBMD measured in the neck of the femur of the non−impaired leg)–0.004(duration of amputation)+0.201. (2)

Adjusted *R* ^2^, SEE and f^2^ were 0.725, 0.079 and 2.64, respectively. The Durbin-Watson was 2.59, indicating that there was no autocorrelation between the residuals. The variance inflation factor was < 1.0 for all the predicting variables showing that multicollinearity between the variables in the model was weak.

## Discussion

To the best of our knowledge, this is the first study investigating the degree of bone demineralization in DXA-measured aBMD at specific skeletal sites (i.e., lumbar spine and hip of both legs) as well as the association between aBMD and some factors associated with bone demineralization in a relatively large (*N* = 40) sample of male athletes with LLA.

The results of the present study showed that athletes with LLA have adequate bone mineralization at the lumbar spine regardless of the level of amputation. In fact, both athletes in the AKA and in the BKA groups have aBMD values at the lumbar spine above the expected range for age according to the ISCD criteria (The International Society for Clinical Densitometry, 2019). These results were expected because of the nature of the considered adapted sports which are practised in positions such that the bones of the lumbar spine are subjected to mechanical forces exerted both by muscle contraction and gravitational loading. In fact, previous findings (Judex and Carlson, 2009; Carter and Hinton, 2014) showed that, further to muscle contraction forces, exercise increases the gravitational loading of the skeleton with a positive influence on bone health. Interestingly, a pattern of bone demineralization similar to that in athletes with LLA showed in this study was also found in other populations suffering from disuse-related osteoporosis like people with SCI (Biering-Sørensen et al., 1988; Biering-Sørensen et al., 1990; Biering-Sørensen et al., 2009). In fact, in people with SCI the greatest bone demineralization after injury was found in the more distal leg sites (i.e., distal femur, proximal tibia and the heel), whereas no bone demineralization occurred in the spine region regardless of the severity of the injury or the activity level (Biering-Sørensen et al., 1988; Morse et al., 2009; McPherson et al., 2014; Peppler et al., 2017). This could be due to the fact that the lumbar spine region continues to be weight loaded after injury, mainly while sitting in a wheelchair (Biering-Sørensen et al., 1988; Jiang et al., 2006). Taken together, these results suggest that the practice of an adapted sport like sitting volleyball, amputee soccer or wheelchair basketball may help in maintaining the aBMD values at the spine and at the hip of the non-impaired leg within the normal ranges despite the amputation.

In agreement with our experimental hypothesis, the results showed that in athletes with LLA, bone demineralization is confined to the hip of the impaired leg. In fact, by considering the non-impaired hip as the control, we observed that the aBMD values were significantly reduced at the impaired hip (by about 27%). Similarly, in people with SCI, the bone mineral content in the femoral neck and shaft was on average 25% lower than normal values, showing that the amount of bone loss after injury is similar to that found in this study in the hip of athletes with LLA. The result of bone demineralization in the impaired leg is also in line with previous literature on non-athletic populations with LLA (Rush et al., 1994; Sherk et al., 2008; Bemben et al., 2017) and suggest that, even if the alterations in bone parameters are confined solely to the impaired-leg, sports practice alone is not enough to limit bone demineralization in such a skeletal site. It is reasonable to assume that it could be due to the nature of the considered adapted sports which are non-impact or low-impact sports, unlike the analogous sports practiced standing (e.g., volleyball, basketball or soccer). Therefore, we infer that the practice of an adapted sport while sitting on a wheelchair or on the playing court could not prevent bone demineralization in the hip of athletes with LLA because in such sport activities the mechanical forces exerted by muscle contraction and gravitational loading are reduced to a minimum. This result is of great importance for physical conditioners and coaches and underlines the importance of implementing standard sport-specific training programs with specific exercise protocols aimed at improving the bone mineralization at the hip of the impaired leg in athletes with LLA.

The results of the present study also highlighted that the amount of bone loss at the hip of the impaired leg experienced by athletes with LLA is affected by the level of amputation. In fact, in agreement with previous literature on non-athletic people with LLA (Sherk et al., 2008), our results showed that, using the hip of the non-impaired leg as a control, athletes with AKA had significantly lower aBMD values at the hip of the impaired leg, with an average percent difference of about 38% (Table 2). Moreover, based on Z-scores, most of them (i.e., 80%; Table 2) have an aBMD value at the hip of the impaired leg below the expected range for age. The combined effect of side differences and amputation level on hip aBMD variables could be explained by both the lack of loading on the amputated limb and the compensatory loading on the contralateral limb (Sherk et al., 2008). In fact, during daily life when people with AKA ambulate while wearing a prosthesis, the loading does not go through the femur but through the soft tissue and probably through the ischial tuberosity (Berger and Fishman, 1997). This might indicate that the compression stresses produced during prosthetic-supported standing and walking alone are insufficient to stimulate lower limb bones even when applied for longer durations. Moreover, during ambulation the non-impaired limb of people with AKA often takes more of the loading (Perry, 2004). On the other hand, athletes with BKA have some biomechanical advantages allowing them to preserve aBMD at the hip of the impaired leg after the injury. These biomechanical advantages are related to the fact that in people with BKA, the loading on the impaired hip is closer to that received by the non-impaired limb while walking (Perry, 2004). What is interesting to highlight is that the results of the present study provide evidence showing that in athletes with AKA the practice of an adapted sport alone is not able to counterbalance the bone loss which occurs in the hip of the impaired leg after an amputation. Accordingly, physical conditioners and nutritionist dealing with bone health of athletes with AKA should consider additional strategies further to sport practice to improve bone mineralization in athletes with AKA.

Another interesting finding of our study was that a greater amount of training and an improved body composition (i.e. lower values of % FM and FM/LM) in the thigh region of the impaired leg were positively associated with higher aBMD values at the hip of the impaired leg despite the level of amputation (Table 3). This result showed that the sport practice may have a role in counterbalancing the bone demineralization at this skeletal site that occurs as a consequence of an amputation. This may be due to the fact that during exercise, bone is subjected to mechanical forces exerted by muscle contraction (Carter and Hinton, 2014). It has been shown (Carter and Hinton, 2014) that skeletal muscle and bone mass and coupled mechanically and, accordingly, they are proportionally related. In fact, in the case of muscular disuse (during inactivity, bed rest or paralysis) the muscle contraction forces are severely reduced as a result of muscle atrophy and this results in site-specific reduction in bone mass and bone strength (Berg et al., 2007). Interestingly, during the face to face questionnaire, we confirmed that, once the post-amputation rehabilitation period ended, no athletes had ever performed specific exercises that target the hip of the impaired leg. On the other hand, these results underlined the importance of prescribing specific exercises aimed at improving the body composition parameters (i.e. reducing fat mass and increasing lean mass) in the thigh of the impaired leg in order to preserve bone mass. This statement is supported by findings in the general population highlighting the potential additive and interactive effects of changes in body composition and strength, with the direct effects of mechanical loading on bone mineral density (BMD) (Snow-Harter and Marcus, 1991; Snow, 1996). In fact, significant correlations of body mass, fat mass, fat-free mass, and strength with total and regional BMD have been found in several studies, with these factors accounting for up to 50% of the variance in BMD (Snow-Harter and Marcus, 1991; Snow, 1996). Accordingly, physical conditioners dealing with athletes with LLA should include exercise training that generates high-intensity loading forces in their plans (i.e., high strain magnitude) as it induces changes in body composition (i.e., a reduction of fat mass and an increase in fat-free mass) and in muscular strength (Kohrt et al., 2004).

Bone needs to be optimally loaded to have a stimulatory effect (Kohrt et al., 2004). In the general population the exercise prescription recommended to help preserve bone health during adulthood involves 30 min–60 min per day of a combination of weight-bearing activities (e.g., stair climbing, jogging) or activities that involve jumping (e.g., volleyball, basketball) for 3–5 sessions per week, and resistance exercise (2–3 sessions per week) with a moderate to high intensity in terms of bone-loading forces (Kohrt et al., 2004). In various analogs to microgravity like bed rest and supine spaceflight, it has been shown (Smith et al., 2008, 2012) that the combination of aerobic and resistance exercise helps in mitigating the net bone loss associated with microgravity. Specifically, this combination is able to promote bone formation, while preventing bone resorption. Moreover, during bed rest, resistance exercise itself may have an independent effect on bone loading and maintain muscle strength during unloading, preserving the capacity to produce muscle-generated force to protect bone (Smith et al., 2008). Similar findings (Smith et al., 2012) were found in astronauts and underlined that the combination of aerobic and resistance exercise during spaceflight, coupled with adequate energy intake and an optimum vitamin D status, increased the concentrations of bone formation markers. The functional electrical stimulation technique is another example of a countermeasure employed to prevent bone demineralization that has been investigated in people with SCI. Scientific data (Abdelrahman et al., 2021) showed that the functional electrical stimulation applied to the paralyzed limbs in people with SCI is able to elicit muscle contractions thereby restoring bone loading. It has also been stated that the efficacy of such interventions is dose-dependent and depends on the session duration (i.e., at least 1 h per day for 5 days per week) and the magnitude of the mechanical loads acting on bone (Bélanger et al., 2000; Frost, 2001; Dudley-Javoroski et al., 2012). More specifically, the magnitude of the load need to be large enough to exceed the remodeling threshold to induce bone formation (Frost, 2001). It has been reported (Bloomfield et al., 1996; Dudley-Javoroski et al., 2012) that BMD was greater in people with SCI who trained at a higher cycling power (i.e., ≥ 18 Watts) (Bloomfield et al., 1996) or in those receiving larger compressive loads (i.e., 150% of body weight) (Dudley-Javoroski et al., 2012) in comparison with those who trained at a cycling power lower than or equal to 12 Watts or those receiving compressive loads lower than or equal to 40% of body weight. According to these studies, the adaptive response of bone to exercise is very complex and depends on the type, intensity, duration, and frequency of the mechanical load. At present, it is not yet possible to describe an exercise program that is proven to help people with LLA to prevent bone demineralization in the hip of the impaired lower limb, because the effect of interventions based on different types of physical activity on bone health as well as quantitative dose-response studies are lacking. However, the scientific data reported above can provide useful guidelines to physical conditioners in planning analogue programs for their athletes aimed at preventing or mitigating bone demineralization.

Another important issue to take into consideration when drawing up a training program to prevent or mitigate bone demineralization in LLA, is that bone responds to site-specific exercises (Winters-Stone and Snow, 2006). In fact, the playing arm of athletes practicing racquet sports is denser and structurally stronger than the non-playing arm (Haapasalo et al., 1994, 1998). Moreover, it has been shown that women who added lower body resistance exercises to their routine (e.g., squats, lunges and calf raises) as well as jump training, actually increased BMD of both the hip and the spine (Winters-Stone and Snow, 2006). On the other hand, women who performed lower body training only, increased hip BMD but not that of the spine (Winters-Stone and Snow, 2006). Accordingly, an optimal intervention for low bone mass at a particular skeletal site should be based on the site specificity of bone loading in order to produce a targeted stimulus to improve BMD. Based on the available literature on the general population (Kohrt et al., 2004; Winters-Stone and Snow, 2006), programs aimed at preventing or mitigating bone demineralization in the hip of the impaired lower limb could for example include: jumping routines varied in type and height, climbing, single leg press, squats and lunges. The challenge for physical conditioners dealing with LLA is to target such exercises according to the specific needs of their athletes and to adapt existing exercises on the basis of the type and severity of amputation.

Interestingly, when assessing the aBMD values at the total proximal femur of the non-impaired leg, the level of amputation and the age at amputation are able to predict about 80% of the total variance for the aBMD values measured at the neck of the proximal femur of the impaired leg. Similarly, when assessing the aBMD values at the neck of the femur of the non-impaired leg, the level of amputation and the duration of amputation can explain almost 80% of the total variance for the aBMD values measured in the neck of the femur of the impaired leg. Both models developed to predict the aBMD values in the impaired leg provided in this study have a useful practical application as they can be used in clinical practice by professionals dealing with the bone health of athletes with LLA when DXA cannot be employed due to the presence of metal implants in the hip of the impaired leg.

This study has some limitations that should be mentioned. First, information on the dietary habits of the participants were not collected. Second, we were not able to collect any hematochemical data like Vitamin D or Calcium levels which are associated with bone mineralization.

In this study there are also some important strengths to underline. First, to the best of our knowledge, this is the first study investigating bone health at specific skeletal sites in athletes with LLA. Second, considering the special population and the available literature dealing with people with LLA, the sample size can be considered large. Third, the sample was homogeneous for several confounding variables like gender, ethnicity and race and several factors associated with bone demineralization (e.g., the absence of any chronic or systemic disease or other physical impairments, apart from the amputation, that might affect body composition or bone metabolism and the non-use of medications that might affect bone metabolism).

In conclusion, this study fills some knowledge gaps in the literature by reporting the DXA-measured aBMD values and Z-scores at specific skeletal sites in athletes with LLA. Furthermore, future research would explore the possible osteogenic impact of other adapted sports on the aBMD values at the hip of the impaired leg, according to the level of amputation. These could include wheelchair sports (e.g., wheelchair basketball or wheelchair rugby), adapted sports practiced in a standing position without wearing prosthesis (e.g., amputee soccer) and adapted sports practiced in a standing position wearing specific prosthesis (e.g., sprint running or long jump).

The results of the present study highlighted that bone demineralization in athletes with LLA is site-specific and is confined at the hip to the impaired leg only. The extent of bone demineralization is influenced by the level of amputation, with athletes with AKA showing more bone loss than athletes with BKA. Even though the practice of an adapted sport and improved body composition in the thigh of the impaired leg may be associated with beneficial bone health in athletes with LLA, in athletes with AKA the practice sport alone is not enough to maintain aBMD values in the impaired hip above the expected range for age. From a practical perspective, physical conditioners and coaches dealing with athletes with LLA should consider alternative strategies, according to the severity of the impairment, which would implement the standard sporting programmes, aimed at improving the bone mineralization at the impaired hip as well as improving body composition in the thigh of the impaired leg. This study also provided two equations able to predict the aBMD values at the hip of the impaired leg of athletes with LLA, when DXA cannot be employed due to the presence of metal implants.

## Data Availability

The raw data supporting the conclusions of this article will be made available by the authors, without undue reservation.
